# Supplementary material for: Dynalign II: common secondary structure prediction for RNA homologs with domain insertions
Source: Nucleic Acids Res. 2014 Nov 21;42(22):13939–48. doi: 10.1093/nar/gku1172 (PMC4267632; doi:10.1093/nar/gku1172)
Supplement: SUPPLEMENTARY DATA [file supp_gku1172_nar-02021-z-2014-File012.zip › manual/RNA_class/html/inherits.html]

RNAstructure: Graphical Class Hierarchy

- Main Page
- Classes

- Class List
- Class Hierarchy
- Class Members

# Graphical Class Hierarchy

Go to the textual class hierarchy

---

Generated on Sun Jul 19 11:06:55 2009 for RNAstructure by 
 1.5.7.1
